# Alveolar macrophages in pulmonary alveolar proteinosis: origin, function, and therapeutic strategies

**DOI:** 10.3389/fimmu.2023.1195988

**Published:** 2023-06-14

**Authors:** Xinmei Huang, Mengshu Cao, Yonglong Xiao

**Affiliations:** ^1^ Department of Respiratory and Critical Care Medicine, Nanjing Drum Tower Hospital, The Affiliated Hospital of Nanjing University Medical School, Nanjing, China; ^2^ Nanjing Institute of Respiratory Diseases, Nanjing, China; ^3^ Department of Respiratory and Critical Care Medicine, Nanjing Drum Tower Hospital Clinical College of Traditional Chinese and Western Medicine, Nanjing University of Chinese Medicine, Nanjing, China; ^4^ Department of Respiratory and Critical Care Medicine, Nanjing Drum Tower Hospital Clinical College of Nanjing Medical University, Nanjing, China

**Keywords:** alveolar macrophage (AM), pulmonary alveolar proteinosis (PAP), granulocyte-macrophage colony-stimulating factor (GM-CSF), pulmonary homeostasis, therapeutic strategies

## Abstract

Pulmonary alveolar proteinosis (PAP) is a rare pulmonary disorder that is characterized by the abnormal accumulation of surfactant within the alveoli. Alveolar macrophages (AMs) have been identified as playing a pivotal role in the pathogenesis of PAP. In most of PAP cases, the disease is triggered by impaired cholesterol clearance in AMs that depend on granulocyte-macrophage colony-stimulating factor (GM-CSF), resulting in defective alveolar surfactant clearance and disruption of pulmonary homeostasis. Currently, novel pathogenesis-based therapies are being developed that target the GM-CSF signaling, cholesterol homeostasis, and immune modulation of AMs. In this review, we summarize the origin and functional role of AMs in PAP, as well as the latest therapeutic strategies aimed at addressing this disease. Our goal is to provide new perspectives and insights into the pathogenesis of PAP, and thereby identify promising new treatments for this disease.

## 1 Introduction

Pulmonary alveolar proteinosis (PAP) is a rare pulmonary disease with a prevalence of 7 cases per million inhabitants worldwide (1, 2). The pathogenesis of PAP involves dysfunction of alveolar macrophages (AMs), resulting from the accumulation of alveolar cholesterol and phospholipids (3). Based on the underlying pathogenesis, PAP is classified into four main categories: primary (90% of cases), secondary (4% of cases), congenital (1% of cases) or unclassifiable. Primary PAP is led by disruption of granulocyte–macrophage colony-stimulating factor (GM-CSF) signaling, resulting in dysfunction of alveolar macrophages (4, 5). Primary PAP can be further classified as autoimmune (caused by elevated levels of anti-GM-CSF antibodies) or hereditary (due to mutations in genes encoding GM-CSF receptors). Secondary PAP occurs as a consequence of factors that reduce the number of AMs and/or impair the function of AMs. These factors include hematologic disorders, medications, environmental exposure, acute silicosis, or immunodeficiency (6). Congenital PAP is caused by genetic variation, including mutations in genes encoding surfactant proteins (SP-A, SP-B or SP-C) or cationic amino acid transport, which disrupts the production of normal surfactant (7). Finally, in some rare cases, patients who exhibit impaired signaling of GM-CSF without any identifiable evidence will be diagnosed with unclassified PAP (8).

Abnormalities of surfactant clearance and dysfunction of alveolar macrophage (AM) are the main causes of PAP (3). Pulmonary surfactant (PS) is a mixture of phospholipids cholesterol (90%) and surfactant proteins A-D (SPs A-D) (10%), which is secreted by lung alveolar type-II (AT-II) cells (9). The lipids of PS are made of DPPC (36%), unsaturated phosphatidylcholine (PC; 32%), phosphatidylglycerol (PG; 8%), cholesterol (7%), other phospholipids (PL; 4%) and other neutral lipids (NL; 3%) (10). The primary function of PS is to prevent alveolar collapse and protect lungs from infections and inflammation, acting as an opsonin as well as directly killing microbes (11). The balances in production, secretion, uptake, recycling and catabolism of PS are crucial for lung homeostasis (12). Seventy percent of spent PS is taken up by AT-II cells and recycled, while the remaining 30% is taken up and degraded by AMs (13).

The functional role of AMs in PAP is crucial, as evidenced by the excessive PS accumulation and the development of PAP in patients lacking functional AMs due to neutralizing autoantibodies against GM-CSF (14), as well as gene deficiencies in CSF2RA or CSF2RB (4, 15). Further studies clarified that intratracheal transplantation of functional AMs corrected PAP of Csf2rb^-/-^ mice (16, 17). Currently, whole lung lavage (WLL) is still the first-line treatment for PAP (18). However, the effectiveness of WLL is limited due to its highly invasive and not curative, and there are no standardized guidelines for the procedure (19). Therefore, exploring the role of alveolar macrophages in PAP may provide new targets and approaches for the treatment of PAP.

In this review, we mainly discuss the current state of knowledge regarding the process of alveolar macrophage development and its role in the pathogenesis of PAP. Our aim is to provide an update on the mechanisms underlying the initiation and progression of PAP. This, in turn, will hopefully aid in the development of effective therapeutic strategies for PAP in clinical settings.

## 2 Origin and development of alveolar macrophages

### 2.1 Origin of alveolar macrophages

There has been a longstanding debate regarding the origin of AMs. Previous studies have suggested that all tissue-resident macrophages, including AMs, originate from circulating monocytes (20–22). However, this theory has been revised during the past decade. Under pathological conditions like osteoarthritis, pancreatic cancer, and steatohepatitis, monocytes can infiltrate target tissues by expressing the C-C chemokine receptor type 2 (CCR2). Therefore, researchers hypothesized that mice lacking Ccr2 would have a lower count of AMs compared to control mice. However, the proportions and numbers of AMs were found to be similar between Ccr2 knockout and control mice, suggesting that circulating monocytes make only a minimal contribution to the AMs pool (23). Moreover, transcriptional and functional data revealed that circulating monocytes rarely serve as precursors of AMs, with different origins, functions, gene expression profiles, and strategies to regulate compartment size (24–26). In line with this, recent studies indicated that alveolar macrophages derive from embryonic monocyte progenitors, which originate from the yolk sac or fetal liver during embryonic development and then migrate into the lung, where they differentiate into alveolar macrophages (25, 27–29). However, it is also feasible that interstitial macrophages derived from circulating monocytes may become precursors of AMs during injury, depletion or explant culture (30–33). Taken together, these studies suggested that alveolar macrophages are capable of dividing, self-renewing, and sustaining themselves without relying on circulating monocytes (23–26).

### 2.2 Factors involved in alveolar macrophages differentiation and maturation

**Figure 1:**
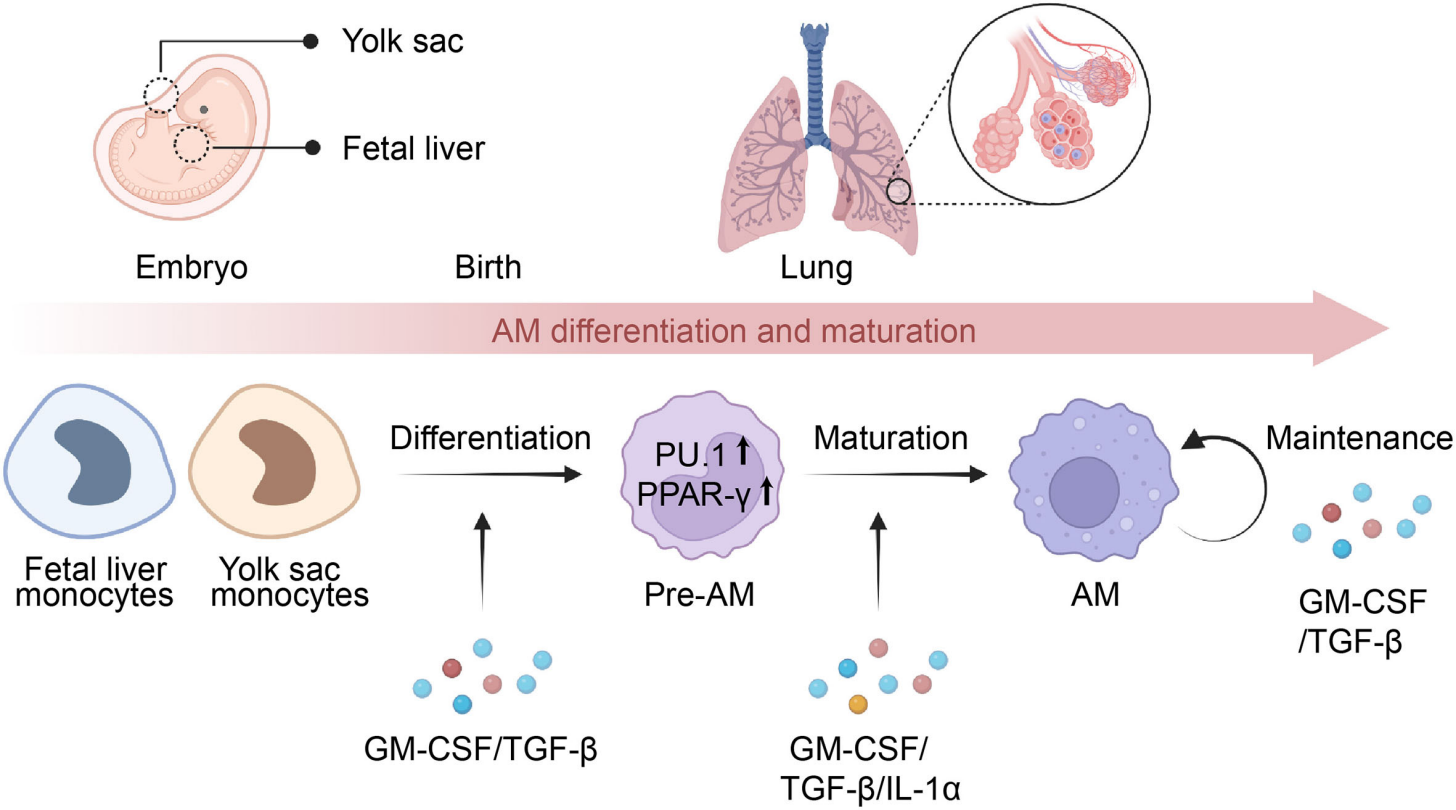
Differentiation and maturation of AMs. Alveolar macrophages (AMs) originate from monocytes present in the yolk sac or fetal liver and later migrate to the lungs, where they develop into pre-AMs and eventually mature into AMs. The process of AM differentiation is facilitated by the presence of GM-CSF and TGF-β, which activate the expression of PU.1 and PPAR-γ, playing a crucial role in determining their identity. Meanwhile, the maturation of pre-AMs relies on the presence of GM-CSF, TGF-β, and IL-1α, leading to transcriptional changes in the signature genes of AMs. Additionally, GM-CSF and TGF-β are essential for the maintenance of mature AMs via an autocrine mechanism.

Several cytokines are involved in AMs differentiation and maturation, including granulocyte-macrophage colony-stimulating factor (GM-CSF), transforming growth factor-beta (TGF-β), and interleukin-1α (IL-1α) (**Figure 1**). GM-CSF serves as a key regulator of AMs terminal differentiation and maintenance, with studies showing that GM-CSF-deficient mice have a significant reduction in the number of mature AMs (15, 34, 35). Further discoveries in patients with rare CSF2RA mutations or anti-GM-CSF autoantibodies confirmed that the unique function of GM-CSF in the lung is fundamentally conserved in humans (4, 36, 37). Notably, GM-CSF triggered the development of AMs by upregulating the expression of PU.1 in AMs (35), and selectively inducing PPAR-γ in fetal monocytes in the lungs (35, 38, 39). In contrast to GM-CSF, although macrophage colony-stimulating factor (M-CSF) serves as a critical cytokine in regulating the development of monocyte and macrophage cells, it has minimal involvement in the differentiation of AMs (40). TGF-β is a multifunctional cytokine that is involved in many biological processes, including immune regulation (41). In contrast to other tissue-resident macrophages, AMs also require TGF-β signaling for development and homeostasis in an autocrine manner (42–44). Further studies indicated that TGF-β signaling leads to transcriptional changes in genes associated with AMs signature (Scgb1a1, Epcam, and Cyp4f18), lipid metabolism (Pparg, Apo-E, Olr1), and the phosphorylation of PPAR-γ (42, 45). IL-1α is another cytokine that has been implicated in AMs differentiation and maturation (46). It was investigated that the lack of IL-1α led to decreased proliferation and maturation of CD11b^low^ alveolar macrophages during granuloma formation, which ultimately resulted in compromised alveolar clearance and the onset of PAP (46).

In addition to cytokines, the transcription factor PU.1 has been shown to be critical for the terminal differentiation of AMs (47–49) (**Figure 1**). Studies utilizing GM-CSF knockout mice have demonstrated that reduced expression of the PU.1 in AMs is associated with decreased expression of CD32, mannose receptor, and macrophage colony-stimulating factor receptor (M-CSFR), which results in the blockade of maturation and differentiation of AMs (47). Moreover, PU.1 is responsible for regulating the constitutive expression of FcγRs and opsonophagocytosis in AMs (49). Notably, GM-CSF signaling is required for the expression of PU.1 in AMs (47, 50). Kruppel-like factor 4 (KLF4) is another transcription factor that is involved in AM differentiation and maturation (51, 52). Further studies have demonstrated that KLF4 promotes AMs polarizing to M2 phenotype through the activation of the downstream STAT6 signaling (52).

## 3 Involvement of alveolar macrophages in PAP progression

### 3.1 Maintenance of surfactant homeostasis by alveolar macrophages in PAP

The pulmonary surfactant (PS) is composed of lipids, including phosphatidylcholine, phosphatidylglycerol, and surfactant protein A-D (53). The vital function of PS is to decrease the surface tension of the alveoli during respiration, which is crucial in preventing lung collapse by reducing the pressure required to inflate the lungs during inhalation (54). Maintaining an appropriate size of the surfactant pool is imperative for proper lung function and is carefully regulated by the balanced processes of surfactant production, secretion, reuptake, recycling, and catabolism within the alveoli (55–57).

**Figure 2:**
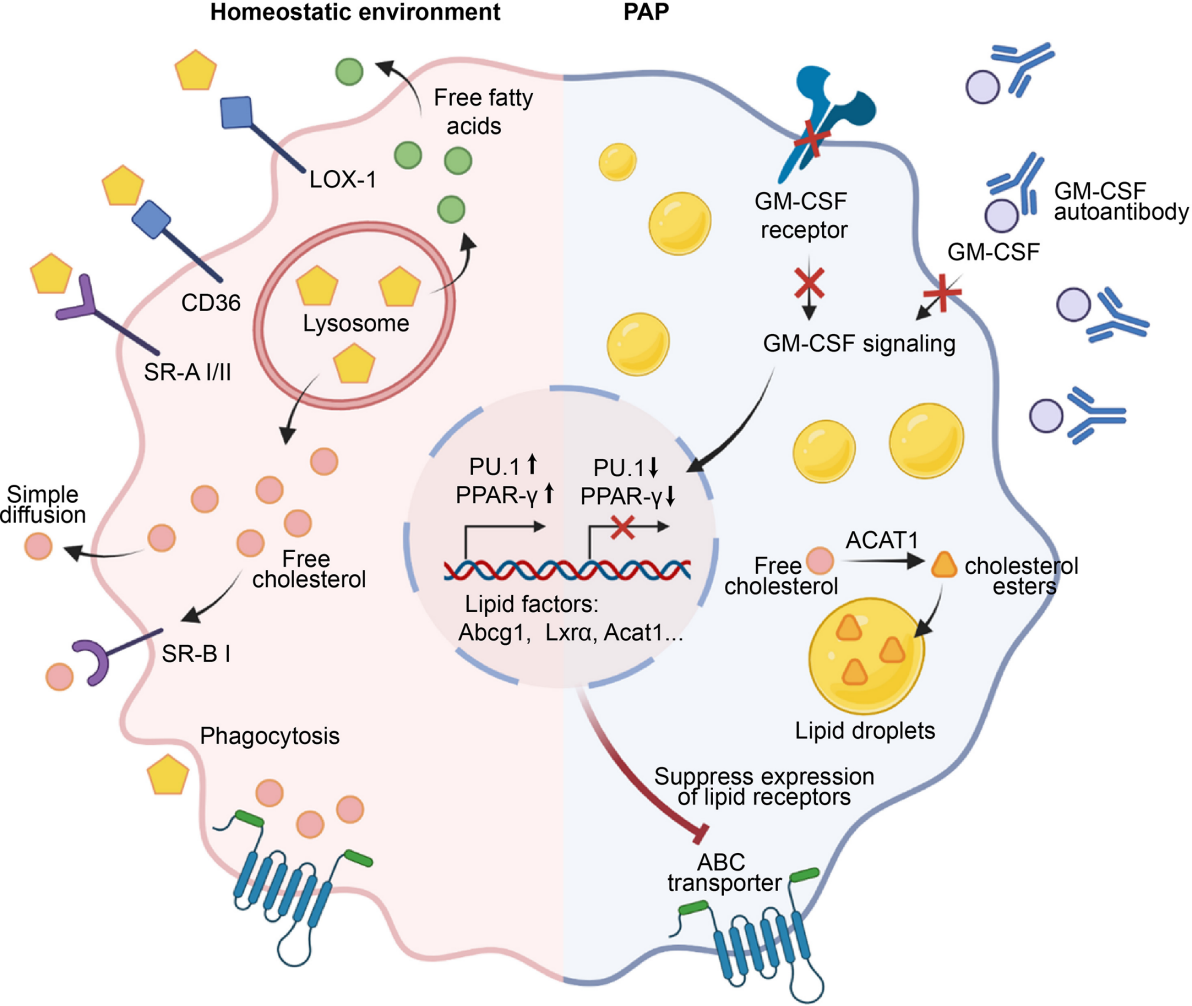
Role of AMs in the maintenance of surfactant homeostasis. Under a homeostatic environment, AMs take up lipids through phagocytosis, macropinocytosis, and scavenger receptors such as LOX-1, SR-A1, SR-B1, and CD36. The engulfed lipids enter lysosomes, where they are degraded into free cholesterol and fatty acids that accumulate in the cell and activate transcription factors such as PPAR-γ, promoting the transcription and expression of downstream lipid factors Abcg1, Lxra, Acat1, etc., and ultimately facilitating the efflux of free cholesterol. In the pathogenesis of PAP, GM-CSF signaling is impaired due to defects in GM-CSF or its receptor, or the presence of anti-GM-CSF autoantibodies. Consequently, this failure to induce the expression of PU.1 and PPAR-γ leads to the downregulation of lipid transporters and dysfunction of AMs. Subsequently, a significant accumulation of free cholesterol occurs within AMs, which is esterified by cholesterol acyltransferase ACAT to form cholesterol esters, stored within lipid droplets. Ultimately, the accumulation of a large number of lipid droplets in the cytoplasm of AMs leads to the generation of foam cells, which results in the deposition of surfactant in the alveoli.

In healthy individuals, pulmonary surfactant is produced and recycled by type II alveolar epithelial cells (AECs), with around 30% of the surfactant being taken up and catabolized by AMs (13, 58). Low-density lipoprotein (LDL), very low-density lipoprotein (VLDL), and oxidized lipoproteins (Ox-L) are taken up by AMs through various mechanisms, including macropinocytosis, phagocytosis, and scavenger receptor-mediated pathways (59) (**Figure 2**). The lipids taken up by AMs are degraded into free cholesterol and free fatty acids in lysosomes. Subsequently, the free cholesterol is re-esterified into cholesterol esters in the endoplasmic reticulum (ER) and stored in the cytoplasm as lipid droplets (60). The accumulation of intracellular cholesterol activates transcription factors such as liver X receptor α and β (LXRα/LXRβ), Retinoid X receptor (RXR), and Peroxisome proliferator-activated receptor α and γ (PPARα and PPARγ), which in turn promote the transcription and expression of lipid transporters ATP binding cassette subfamily A member 1(ABCA1) and ATP-binding cassette transporter G1(ABCG1) (61–64). These transporters subsequently regulate the efflux of free cholesterol and the maturation of HDLs (65). In addition, the efflux of free cholesterol is facilitated by simple diffusion or SR-BI-mediated facilitated diffusion (66).

Pulmonary alveolar proteinosis (PAP) is characterized by a reduction in AMs-mediated surfactant clearance, resulting in the formation of dysfunctional foamy AMs and accumulation of alveolar surfactant (67, 68). GM-CSF plays a key role in surfactant clearance of AMs, as evidenced by the development of hereditary PAP in patients with deficiencies in either CSF2RA or CSF2RB resulting in a lack of AMs (4, 69), and the development of autoimmune PAP in patients with non-functional AMs due to neutralizing auto-antibodies against GM-CSF (70). The esterified and free cholesterol are mainly accumulated by AMs in PAP, while the surfactant component displays a higher proportion of cholesterol (71). Mechanistically, the primary defect in cholesterol clearance caused by disruption of GM-CSF signaling leads to a secondary reduction in both surfactant uptake and clearance (71) (**Figure 2**). Moreover, disruption of GM-CSF signaling also caused a decrease in mRNA levels of several cholesterol homeostasis factors that are critical to AMs, including Abcg1, Lxrα, Acat1, Neutral cholesterol ester hydrolase 1(Nceh1), and Lipase A (Lipa) (71, 72). In line with this, replenishing PPAR-γ in AMs of GM-CSF knockout mice results in a decrease in intracellular lipid accumulation and an increase in cholesterol efflux activity by upregulating ABCG1 (73). Similar studies showed that PPAR-γ is implicated in the mitigation of foamy AMs formation by reducing the expression of ABCG1, CD36, Oxidized low-density lipoprotein receptor 1(Olr1), Fatty acid-binding proteins 1(Fabp1), Fabp4, and Cell Death Inducing DFFA Like Effector C(Cidec), which play crucial roles in the uptake, transport, storage, and processing of lipids (38, 74–76). Interestingly, lipid metabolism factors ABCA1 and LXRβ are inadequate in preventing surfactant accumulation in AMs (75). In addition to PPAR-γ, another crucial transcription factors regulated by GM-CSF is PU.1, which is known to modulate the expression of lipid transporters ABCA1 and ABCG1 (72, 76). Collectively, these studies indicate that GM-CSF signaling is necessary for cholesterol clearance in AMs and identify impaired cholesterol clearance as the main AMs abnormality underlying the development of PAP.

### 3.2 Immunological functions of alveolar macrophages in PAP

The alveolar immune microenvironment is a complex, multi-cellular system consisting of alveolar macrophages, dendritic cells, lymphocytes, and epithelial cells (77). These cells collaborate to maintain pulmonary homeostasis and defend against inhaled pathogens and environmental insults (78). Alveolar macrophages, as the predominant phagocytic cells in the lungs, play an essential role in the removal of inhaled particles and pathogens (79). Additionally, they release cytokines and chemokines that orchestrate the recruitment and activation of other immune cells at the site of infection (80).

**Figure 3:**
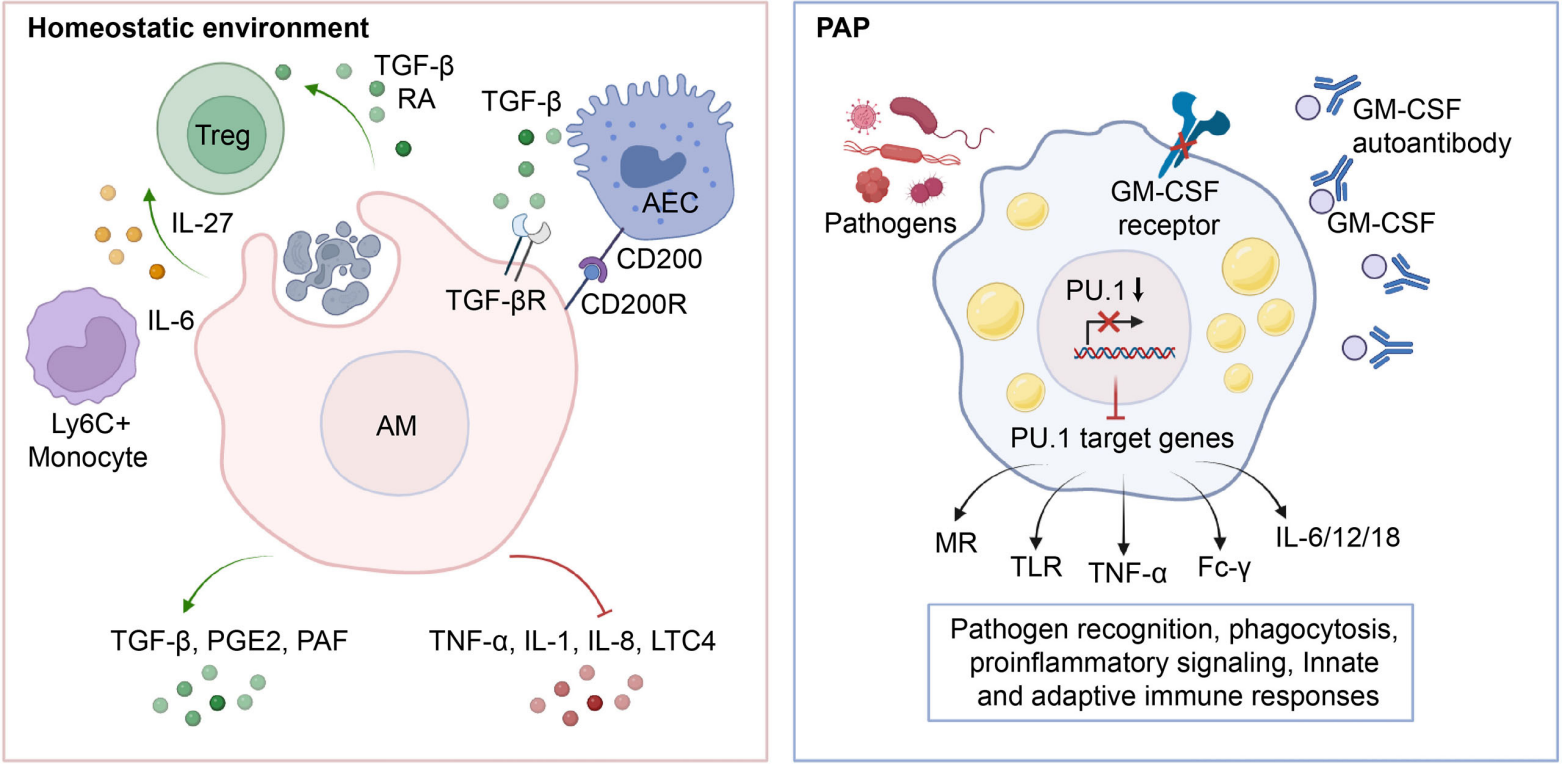
Role of AMs in the maintenance of immunological homeostasis. In physiological conditions, AMs phagocytose apoptotic cells, leading to an increase in the production of anti-inflammatory cytokines such as TGF-β, PGE2, and PAF, which in turn inhibits the release of pro-inflammatory cytokines TNF-α, IL-1, IL-8, and LTC4 into the microenvironment. Additionally, AMs induce Treg differentiation by secreting TGF-β, RA, IL-27, etc., and maintain lung immune homeostasis through crosstalk with AECs. In PAP, the impairment of GM-CSF signaling leads to downregulation of PU.1 expression, which in turn reduces the transcription and expression of downstream target genes such as MR, TLR, TNF-α, Fc-γ, and IL-6/12/18. This inhibition of antigen recognition, phagocytosis, pro-inflammatory signaling, and related innate and adaptive immune responses in AMs ultimately predisposes PAP patients to secondary infections.

Under physiological conditions, AMs demonstrate heightened phagocytic activity, low inflammatory cytokine production, and overall suppression of inflammation and adaptive immunity (80, 81) (**Figure 3**). AMs facilitate the resolution of inflammation by phagocytosing apoptotic and necrotic cells (a process known as efferocytosis), which in turn prevents the release of proinflammatory cytokines such as tumor necrosis factor alpha (TNF-α), Interleukin-1(IL-1), IL-8, and leukotriene C4 in the alveolar microenvironment (82–84). Consistent with this, efferocytosis prompts AMs to secrete anti-inflammatory cytokines such as TGF-β, prostaglandin E2 (PGE2), and platelet-activating factor (PAF), thereby preventing the onset of unnecessary inflammatory responses (85–87). Apart from its role in efferocytosis, an alternative mechanism through which AMs inhibit inflammation is by activating the regulatory T cells (Treg) immune response (88). It was demonstrated that the isolation of mouse-derived AMs, which were subsequently stimulated with ovalbumin and co-cultured with antigen-specific naive CD4^+^ T cells, led to generation of Foxp3^+^ Treg cells (89). Notably, this functional role of AMs was partially reversed by either inhibiting the binding of retinoic acid (RA) to its receptor (RAR), or by impeding the signaling of TGF-β (89). In addition to directly affecting Treg cell conversion by influencing the components of RA and TGF-β, AMs may also initiate the generation of Tregs through an indirect pathway (90–92). In the context of respiratory viral infections, AMs generate the immunoregulatory cytokine IL-27 via the induction of IL-6, which in turn recruits Ly6C^+^ monocytes and facilitates the local differentiation of Tregs (92). Communication with the alveolar microenvironment also modulates the immunological functions of AMs. The binding of CD200 and TGF-β, present on the cell membrane of alveolar epithelial cells, to their respective receptors, CD200R and TGF-βR, expressed on AMs, functions as a negative regulator of AM pro-inflammatory activity (93–95).

In patients with PAP, the lack of GM-CSF signaling impairs the immunological functions of AMs and neutrophils, making them more vulnerable to superimposed infections caused by fungal pathogens, nocardia, cytomegalovirus, pneumocystis carinii, anaerobes, and mycobacteria (96–102). Generally, up to 13% of patients with PAP may experience secondary pulmonary infections, with fungal infections being more commonly diagnosed and mycobacterial infections having the lowest mortality rate (99). About 20% of deaths in PAP patients are caused by secondary infections (103). Similarly, AMs from GM-CSF-deficient mice exhibit reduced phagocytosis, pathogen killing, expression and release of pro-inflammatory cytokines (35). Mechanistically, the impaired maturation and function of AMs resulting from GM-CSF signaling deficiency is the primary factor of secondary infections in PAP (50) (**Figure 3**). It was found that GM-CSF promotes the uptake and clearance of adenovirus by AMs through a PU.1-dependent transcriptional program that redirects virion trafficking from the nucleus to lysosomes (104). Moreover, GM-CSF also regulates the production of genes that facilitate multiple immune and non-immune functions by stimulating the expression of PU.1 in AMs (50). It was demonstrated that the expression of PU.1 in GM-CSF-deficient AMs significantly increase the expression of mannose receptor (MR) and Toll-like receptor 2/4 (TLR-2/4), which are involved in the phagocytic internalization of microorganisms and pro-inflammatory signaling responses (35, 105). In line with this, the expression of PU.1 in GM-CSF-deficient AMs mediates a dose-dependent increase in the release of TNF-α and IL-6 (35). In addition, GM-CSF/PU.1 signaling orchestrates the molecular crosstalk between innate and adaptive immunity by modulating the production of IL-12, IL-18, and IFN-γ, consisting with the regulation of AMs phagocytosis via Fc-γ receptors (49). Together, these studies suggest that AMs have a critical function in preserving pulmonary immunological equilibrium in PAP patients, underscoring the essentiality of meticulous monitoring for infectious complications.

## 4 Therapy of PAP by targeting alveolar macrophages

Whole-lung lavage (WLL) is a standard treatment for patients of PAP, which generally improves pulmonary function in the majority of patients (106). However, due to the impaired function of AMs and the recurrence of surfactant accumulation, repeated treatments are often necessary (107). Therefore, restoring normal AM function to maintain pulmonary lipid and immune homeostasis is a fundamental approach for the treatment of primary PAP (108). In the treatment of secondary PAP, the primary approach is to address the underlying primary disease or remove the causative factors, with adjunctive whole lung lavage (WLL) being an additional option to enhance therapeutic efficacy in severe cases (109, 110). Furthermore, gene therapy-induced restoration of normal AM function also serves as a therapeutic option for hereditary PAP (111). In this section, we will discuss the applications of GM-CSF administration, rituximab, pharmacotherapies that restore lipid homeostasis in AMs, AM transplantation, and induced pluripotent stem cell (iPSC)-derived AMs in the treatment of PAP.

### 4.1 GM-CSF

The administration of GM-CSF is a promising therapeutic strategy for autoimmune PAP, as it neutralizes the anti-GM-CSF antibodies and restores AM function. However, the effectiveness of GM-CSF therapy in secondary PAP remains to be established (112). A number of case series have reported that administering exogenous GM-CSF therapy has led to clinical improvement in patients with PAP (**Table 1**). Recombinant GM-CSF is commonly administered through subcutaneous injection or inhalation, with various dosing regimens, including low-dose treatment (125 μg/day) and high-dose treatment (250 or 300 μg/day), etc (113–116, 118, 120–123). Therapeutic response of GM-CSF administration was evaluated via disease severity (alveolar arterial gradient (A-a) Do_2_, oxygenation, etc.), pulmonary function tests, serum biomarkers of PAP, HRCT score, respiratory symptom scores, etc. The majority of the case series documented a response rate of 30-60%, with remission of respiratory symptoms and improvement in pulmonary function (113–116, 118, 120–123). Intriguingly, Campo et al. demonstrated that inhaled recombinant GM-CSF was well tolerated and effective in a PAP, and combination therapy proved to be more effective than WLL alone (119). Moreover, Tazawa et al. conducted a study in 3 successfully treated patients with idiopathic PAP, which found that the cell number, expressions of surface mannose receptor and PU.1, and phagocytic ability of AMs were all restored to control levels (124). Notably, the treatment resulted in a significant reduction in the neutralizing capacity and the levels of anti-GM-CSF autoantibodies in BALF of patients. Similarly, other studies also revealed that the administration of GM-CSF restored the morphology and adhesive function of AMs, upregulated the expression of GM-CSF receptor and PU.1, and improves lung clearance in PAP (47, 117, 125).

**Table 1:** Relevant clinical data on therapy of PAP by GM-CSF administration.

Authors	Sample	Dosage, regimen, duration	Endpoints of efficacy	Efficacy data
Seymour et al., 2001 (113)	N=14	5 μg/kg/day, 6-12 weeks, Subcutaneous injection	Alveolar gas exchange, dynamic imaging of lung abnormalities, exercise capacity	43% response rate
Venkateshiah et al., 2006 (114)	N=25	250 μg/day for month 1, 5 μg/kg/day for month 2, 9 μg/kg/day for month 3, Subcutaneous injection	Reduction in oxygenation>10 mmHg	48% response rate
Wylam et al., 2006 (115)	N=12	250 μg/day every other week for 64 weeks in responders, 250 μg/day every other week for 12 weeks and then 500 μg/day for up to 64 weeks in non-responders to the initial dose, Inhalation	Radiological response (clearance of abnormalities), Clinical and pulmonary function improvement	11 patients reported positive response
Tazawa et al., 2010 (116)	N=39	High-dose therapy (250 μg Days 1–8, none on Days 9–14; 6 cycles); low-dose therapy (125 μg Days 1–4, none on Days 5–14; 6 cycles), Inhalation	Reduction in A–aDO_2_>10 mmHg, pulmonary function tests, serum biomarkers of PAP	62% response rate
Ohashi et al., 2012 (117)	N=19	Total dose 10.5-21 mg, duration 12-24 weeks, Inhalation	Improvement in the surfactant clearance	Total protein and surfactant protein-A (SP-A) were significantly decreased in high responders
Papiris et al., 2014 (118)	N=6	250 μg/day given 4 days on and 4 days off for as long as necessary, Inhalation	Absence of symptoms, oxygen desaturation less than 4% at the 6MWT, and significant radiographic reduction of the infiltrates	6 patients responded to treatment, 3 patients maintained remission at the lowest effective dose
Campo et al., 2016 (119)	N=18	250 μg/day every other week for 12 weeks followed by 250 μg/day on 2 consecutive days every 2 weeks for 6 months, Inhalation	Pulmonary function (DLCO%, FVC%, TLC%, FEV1%), PaO_2_ and alveolar-arterial oxygen gradient	Significant improvement in pulmonary function, PaO_2_ and alveolar-arterial oxygen gradient
Tazawa et al., 2019 (120)	N=64	250 μg/day for 7 days, 25 cycles, Inhalation	The change in the alveolar–arterial oxygen gradient between base line and week 25	-0.45 vs 0.17 mmHg, P=0.02
Tian et al., 2020 (121)	N=36	250 μg/day for month 1-3, 125 μg/day for month 4-6, Inhalation	The change of A-aDO_2_ from the baseline, pulmonary function (DLCO%, TLC%)	No significant differences in A-aDO2, pulmonary function improved (P = 0.010, 0.027)
Zhang et al., 2020 (122)	N=55	75 μg/day for month 1, 12 months follow up period, Subcutaneous injection	Cure (absence of baseline imaging abnormalities, normal lung function, no symptoms, normal exercise capacity); Improvement (significant improvement of the above measures)	31% cure, 29% Improvement
Trapnell et al., 2020 (123)	N=138	300 μg/day for 7 days, either continuously or intermittently (every other week) for 24 weeks, Inhalation	The change from baseline in the alveolar–arterial difference in oxygen concentration (A-aDo_2_) at week 24	Patients receiving continuous molgramostim showed greater improvement compared to those receiving placebo

### 4.2 Rituximab

Rituximab is a chimeric monoclonal antibody that specifically recognizes the B-lymphocyte antigen CD20 (126). It has been previously used to treat CD20^+^ B-cell lymphoma, as well as autoimmune diseases such as systemic lupus erythematosus and rheumatoid arthritis (126, 127). Expanding on this, Rituximab has also been utilized as a B-cell depleting agent in order to eradicate anti-GM-CSF autoantibodies and subsequently serve as a therapeutic intervention for PAP. Multiple studies have reported positive outcomes in terms of improvement in lung function and imaging variables in cases of autoimmune PAP following treatment with Rituximab, as observed in both case series and isolated cases (128–131) (**Table 2**). In addition, the administration of Rituximab therapy led to a consistent reduction in both CD19^+^ B-cells in peripheral blood and anti-GM-CSF autoantibodies in BALF (131). Mechanistically, Rituximab therapy ameliorates the lipid metabolism of AM in patients with PAP, as demonstrated by the upregulation of key lipid metabolism molecules, including PPAR-γ, ABCG1, and lysosomal phospholipase A2 (LPLA2), a critical enzyme for surfactant degradation (132). Furthermore, Oil Red O staining indicates a reduction in intracellular lipid droplets in AMs, leading to an increase in free cholesterol in BALF and maintaining lipid homeostasis in the alveoli (132).

**Table 2:** Relevant clinical data on therapy of PAP by Rituximab.

Authors	Sample	Dosage, regimen, duration	Endpoints of efficacy	Efficacy data
Borie et al., 2009 (129)	N=1	1,000 mg intravenous infusion on day 1 and day 15	Improvements in lung function tests (DLCO %, Alveolar–arterial gradient at rest mmHg, six-minute walk test) and lung CT images	A clear improvement on the last follow-up visit 12 months
Kavuru et al., 2011 (131)	N=10	1000 mg, two doses with a 15-day interval between each infusion, intravenous infusions	Partial arterial oxygen pressure and alveolar-arterial gradient in room air, Lung function, HRCT scans	70% response rate
Soyez et al., 2018 (130)	N=13	12 patients were treated with rituximab (1000mg) with two infusions at day 0/14, with 3 patients receiving a third infusion at month 6/9/12 respectively, while 1 patient received only one infusion	Reduction in A–aDO_2_>10 mmHg, Disease severity score (DSS)	No patients showed improvement, 4 patients (30%) presented a significant decrease of alveolar-arterial difference in oxygen
Bird et al., 2022 (128)	N=1	Two doses of 1 g of rituximab were administered intravenously, separated by 14 days	Improvements in arterial oxygenation, respiratory membrane gas diffusion, six-minute walk test and radiological findings	A significant clinical response

### 4.3 Pharmacotherapies that restore lipid homeostasis in alveolar macrophages

Currently, the pharmacotherapies utilized to restore lipid homeostasis in AMs comprise Pioglitazone and Statins. In the development of PAP, the absence of key molecules PPAR-γ and ABCG1 in AM is a crucial factor contributing to the dysregulation of cholesterol transport (75). In line with this, restoration of PPAR-γ signaling leads to a subsequent upregulation of ABCG1 expression, which facilitates the efflux of intracellular cholesterol in AMs (73). Pioglitazone is an agonist of PPAR-γ, which has been historically employed for the treatment of type 2 diabetes and Alzheimer’s disease (133, 134). Recent research utilizing Csf2rb^-/-^ PAP mice has demonstrated that pioglitazone can increase the expression of ABCG1, enhance cholesterol efflux in AMs, and alleviate pulmonary symptoms in PAP mice (135). A comparable outcome was documented in an autoimmune PAP patient that was unresponsive to conventional first-line therapies but demonstrated improvement of lung function with pioglitazone treatment (30 mg taken once daily) (136). The clinical study investigating the efficacy of pioglitazone in treating PAP is underway (NCT03231033) (136).

Statin is primarily used to reduce the risk of cardiovascular diseases by lowering cholesterol levels in the blood (137). Notably, a study involving two autoimmune PAP patients who were unresponsive to WLL treatment found that statins could significantly improve the patients’ clinical symptoms, lung function, and radiological features, as well as reduce the intracellular cholesterol levels in AMs (138). In Csf2rb^−/−^ mice, statin treatment also promoted cholesterol efflux in AMs and alleviated disease symptoms (138). In addition, a prospective real-world observational study evaluated the therapeutic efficacy of statins on 40 non-hypercholesterolemic PAP patients (139). 65% of the patients showed a treatment response, characterized by an increase in PaO_2_, DLCO%, and a decrease in disease severity score (DSS) and radiographic abnormalities (139). The cutoff dose was 67.5 mg daily, with a corresponding specificity of 64.3% and sensitivity of 96.2% (139).

### 4.4 Transplantation of gene-corrected alveolar macrophages and induced pluripotent stem cell-derived alveolar macrophages

In addition to pharmacological treatments aimed at restoring AM function, direct transplantation of gene-corrected AMs or iPSC-derived AMs has also shown promise in the treatment of hereditary PAP. A study was conducted using lentiviral-mediated overexpression of the Csf2ra gene to prepare gene-corrected AMs, which were then transplanted into Csf2ra^-/-^ mice. The results showed that the transplantation improved AM function, restore lipid homeostasis and surfactant metabolism, and ameliorated the cytological, histological, and biomarker abnormalities associated with PAP (140). To enable the translation of gene-corrected AM therapy to PAP patients, a self-inactivating (SIN) lentiviral vector expressing a codon-optimized human CSF2RA-cDNA driven by an EF1α short promoter (Lv.EFS.CSF2RAcoop) was created. Results indicated that Lv.EFS.CSF2Racoop restored GM-CSF signaling in AMs by reconstituting GM-CSF receptor expression, and did not elicit negative effects in the target cells (141). Similarly, by using TALEN-mediated genetic integration, a codon-optimized CSF2RA transgene was inserted into the AAVS1 locus of iPSCs derived from hereditary PAP patients. This restored GM-CSF receptor functionality and corrected the disease phenotype of monocytes/macrophages derived from hereditary PAP iPSCs *in vitro (* 111). Additionally, it has been demonstrated that human iPSC-derived macrophages can be transplanted into the lungs and differentiate into AMs, leading to a decrease in alveolar proteinosis in a humanized PAP model (142, 143).

## 5 Conclusion

AMs are a crucial type of immune cell in the pulmonary microenvironment, which are mainly derived from monocytes present in the yolk sac or fetal liver, and later migrate to the lungs, where they develop into pre-AMs and eventually mature into AMs. GM-CSF and TGF-β, along with their downstream signals PU.1 and PPAR-γ, play a critical role in the differentiation and maintenance of AMs. In the pathogenesis of PAP, GM-CSF signaling is impaired due to defects in GM-CSF or its receptor, or the presence of anti-GM-CSF autoantibodies, which fail to induce the expression of PU.1 and PPAR-γ, resulting in abnormal development and function of AMs. Ultimately, the cytoplasmic accumulation of lipid droplets in AMs results in the development of foam cells, which leads to the deposition of surfactant in the alveoli. Additionally, AMs are also critical in maintaining pulmonary immune homeostasis. In PAP, the impairment of GM-CSF signaling leads to the downregulation of PU.1 expression, which in turn reduces the transcription and expression of downstream immune-related target genes. This leads to abnormal pulmonary innate and adaptive immune responses, ultimately making PAP patients susceptible to secondary infections. Currently, exogenous GM-CSF has been reported as a treatment option for autoimmune PAP, while its utility in other forms of PAP remains unknown. Moreover, rituximab, pharmacotherapies targeting lipid homeostasis in AMs, AM transplantation, and induced pluripotent stem cell (iPSC)-derived AMs have also been utilized for PAP treatment. The objective of these therapies is to directly or indirectly restore normal differentiation and function of AMs.

Although significant progress has been made in comprehending PAP in the last few decades, several crucial questions remain unanswered and will motivate future research on the etiology, pathogenesis, and therapies. Despite notable advancements in the etiology research of PAP, the cause of anti-GM-CSF autoantibody production remains unknown. In addition, although the pathogenic mechanism of abnormal AM function resulting from GM-CSF signaling deficiency has been elucidated, the downstream key effectors and mechanisms involved in lipid and immune homeostasis are still unclear. It is worth noting that currently, AM-targeted PAP therapies, such as GM-CSF administration, rituximab, and AM transplantation, have been demonstrated to be effective and safe. Nonetheless, it is crucial to underscore that these therapies have not yet obtained regulatory approval. In addition, there is still a lack of established therapeutic guidelines and consensus, and some promising treatment strategies are still undergoing clinical validation. We hope that this review will provide perspectives on understanding the function of AMs in PAP and developing new therapeutic strategies.

## Author contributions

Manuscript drafting and figures & tables design: XH. Writing-review and editing: MC and YX. Supervision: MC and YX. Funding acquisition: MC and YX. All authors contributed to the article and approved the submitted version.

## Glossary

**Table:**

ABCA1	ATP binding cassette subfamily A member 1
ABCG1	ATP-binding cassette transporter G1
AECs	Alveolar Epithelial Cells
AM	Alveolar Macrophage
Apo-E	Apolipoprotein E
AT-II	Alveolar Type-II cells
BALF	Bronchoalveolar Lavage Fluid
CCR2	C-C chemokine Receptor type 2
Cidec	Cell Death Inducing DFFA Like Effector C
DSS	Disease Severity Score
EpCAM	Epithelial Cell Adhesion Molecule
ER	Endoplasmic Reticulum
Fabp	Fatty acid-binding proteins
GM-CSF	Granulocyte–Macrophage Colony-Stimulating Factor
IL-1α	Interleukin-1α
iPSC	induced Pluripotent Stem Cell
KLF4	Kruppel-Like Factor 4
LDL	Low-Density Lipoprotein
Lipa	Lipase A
LPLA2	lysosomal phospholipase A2
LXRα/LXRβ	liver X receptor α and β
M-CSFR	Macrophage Colony-Stimulating Factor Receptor
M-CSF	Macrophage Colony-Stimulating Factor
MR	Mannose Receptor
Nceh1	Neutral cholesterol ester hydrolase 1
Olr1	Oxidized low-density lipoprotein receptor 1
Ox-L	Oxidized Lipoproteins
PAF	Platelet-Activating Factor
PAP	Pulmonary Alveolar Proteinosis
PGE2	Prostaglandin E2
PPAR	Peroxisome proliferator-activated receptor
PS	Pulmonary Surfactant
RA	Retinoic Acid
RXR	Retinoid x receptor
SCGB1A1	Secretoglobin family 1A member 1
TGF-β	Transforming Growth Factor-beta
TLR	Toll-like Receptor
TNF-α	tumor necrosis factor alpha
VLDL	Very Low-Density Lipoprotein
WLL	Whole Lung Lavage.
